# Effect of temperature on the oxidation of Cu nanowires and development of an easy to produce, oxidation-resistant transparent conducting electrode using a PEDOT:PSS coating

**DOI:** 10.1038/s41598-018-28744-9

**Published:** 2018-07-13

**Authors:** Dedi Mardiansyah, Trevon Badloe, Kuwat Triyana, Muhammad Q. Mehmood, Niloufar Raeis-Hosseini, Yoonkyung Lee, Harsojo Sabarman, Kyunghoon Kim, Junsuk Rho

**Affiliations:** 1 Department of Physics, Universitas Gadjah Mada, Yogyakarta, 55281 Indonesia; 2 Department of Physics Education, Universitas Pasir Pengaraian, Riau, 28558 Indonesia; 3 Department of Mechanical Engineering, Pohang University of Science and Technology (POSTECH), Pohang, 37673 Republic of Korea; 4 Nanomaterials Research Group, Universitas Gadjah Mada, Yogyakarta, 55281 Indonesia; 5 Department of Electrical Engineering, Information Technology University of the Punjab, Lahore, 54000 Pakistan; 6 Department of Chemical Engineering, Pohang University of Science and Technology (POSTECH), Pohang, 37673 Republic of Korea; 7 School of Mechanical Engineering, Sungkyunkwan University, Suwon, 16419 Republic of Korea

## Abstract

Oxidation can strongly influence the performance of Cu nanowires (CuNWs) by decreasing their conductivity. Here, we identify and investigate a way to prevent the oxidation process of CuNWs to maintain the high conducting performance of CuNWs as transparent electrodes. CuNWs were synthesised using an aqueous method. We prepared several temperature treatments (from 0–300 °C) to represent oxidation of CuNWs in different environments, to study the oxidation process and changes in morphology in detail. Depending on the temperature, smooth and uniform CuNWs exposed to oxidation produced rough Cu_2_O and CuO nanowires. We then suggest a method of protecting nanowires from oxidation, using the Mayer rod coating method to apply a layer of PEDOT:PSS to a transparent conducting film of CuNWs. The result indicates that this method of protection can protect the film, and maintain a stable, and constant resistance over of time, without effecting the excellent conductivity properties of pure CuNWs.

## Introduction

Development of transparent conducting electrodes has attracted attention in recent years. Indium tin oxide (ITO) had been the dominant material used up to now due to its excellent transmittance (90%) and conductance (10S)^1^. However, ITO is brittle and is of scarce supply in nature. An alternative to ITO is to use silver (Ag) nanowires (NWs) which have excellent transmittance, conductance and flexibility but is also unfortunately not very abundant.

Copper (Cu) has superb electrical and optical performance, comparable to that of other metals such as silver and indium, but has the advantage of being thousands of times more abundant. Due to its relatively plentiful supply, low cost and high electrical and optical performance, one dimensional (1-D) copper has drawn lots of interest of late and the oxidation of Cu nanowires (CuNWs) can be used to create Cu_2_O and CuO nanowires with vastly different electrical properties. CuNWs are simply conductors, while Cu_2_O and CuO nanowires are p-type semiconductors with narrow direct band gaps of ~2.1^2^ and ~1.2 eV^3^, respectively. These oxidised CuNWs have various potential applications such as, transparent conductors^4,5^, gas sensors^6^, solar cells^7^, electronic devices^8^ and catalysts^9,10^.

The fabrication of Cu nanostructures has improved tremendously in recent years by utilising various techniques such as, hydrothermal treating^11^, templates^12^, electrochemical deposition^13^, electrodeposition^14^ and aqueous solutions^15,16^. Up to now, research has been focused on the synthesis of CuNWs with the goal of increasing the aspect ratio. However, oxidation effects on CuNWs structures have not yet been clearly discussed. Lee *et al*. (2015), reported the effects of post-annealing treatment on the microstructural evolution and quality of Cu(OH)_2_ nanowires^17^. Nunes *et al*. (2015), reported the oxidation of CuNWs to Cu_2_O nanowires with a comparison between microwave irradiation and furnace annealing under atmospheric conditions^18^. Won *et al*. (2014), reported annealing-free fabrication of highly oxidation-resistive copper nanowire composite conductors^19^. More investigation into the oxidation process of CuNWs is still required. Here, we study the effects of oxidation on CuNWs structures in detail, with a range of temperatures from 0 °C to 300 °C, to produce Cu_2_O and CuO nanowires.

It has been shown that the oxidation of CuNWs is influenced by the temperature^20^. In this work, first, we controlled the temperature and therefore the oxidation of CuNWs to create Cu_2_O and CuO nanowires. The products of oxidation at four different temperatures were investigated with XRD, FTIR and SEM-EDX. This information is necessary for researchers developing CuNWs based applications, specifically the design and synthesis of appropriate materials with desirable properties, such as high surface area, conductivity, and resistance, chemical and structural stability, and cheap mass production.

The development of flexible, transparent, conducting electrodes is of great interest^21^. There are many applications for transparent, conducting electrodes such as, electronic devices^22^, solar cells^7^, organic light emitting diodes (OLEDs)^23^, and sensors^24^. Some of the weaknesses of CuNW films are, rapid oxidation, complicated synthesis process and long reaction times. Here we investigate the prevention of oxidation of CuNWs. There are limited reports about protecting CuNWs from oxidation. One such study used polyvinyl pyrrolidone (PVP) to protect the CuNWs^4^. Using PVP achieves the goal of protecting the nanowires from oxidation, but has the inherent drawback of decreasing conductivity since PVP is an insulating polymer. In recent years, ethylenediamine (EDA) has been used as a protective coating for CuNWs^25^, but this technique has the same problem of reduced conductivity, limiting usage in future applications. Here, we report a method of preventing oxidation of CuNWs without hindering conductivity performance. The nanowires are protected from oxidation by using the Mayer Rod coating method to deposit a protective layer of the conductive compound Poly(3,4-ethylenedioxythiophene) polystyrene sulfonate (PEDOT:PSS) on them. This can prevent oxidation without decreasing conductivity, overcoming the biggest problem with previous methods with a simple process of coating.

## Results

### Oxidation process of Cu nanowires

**Figure 1:**
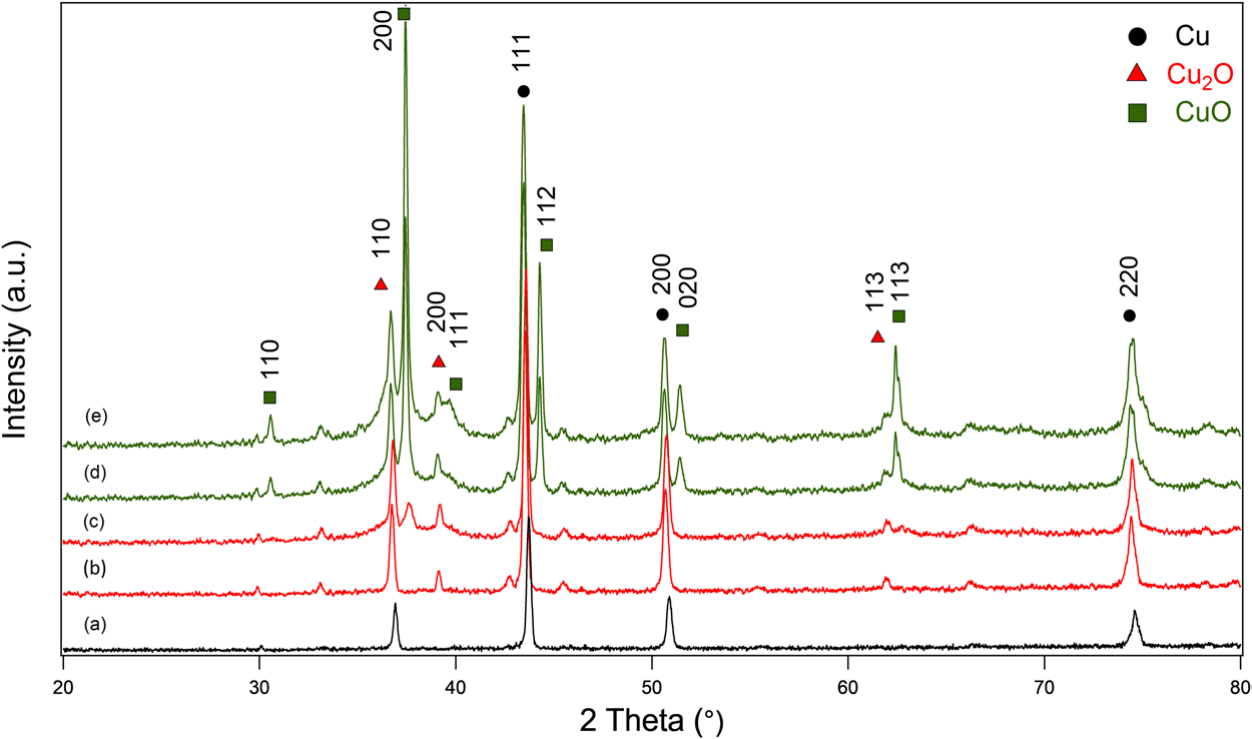
XRD patterns of Cu nanostructures. (**a**) A CuNWs (JCPDS 04-0836), (**b**) CuNWs kept at 0 °C for 8 hours, (**c**) CuNWs kept at RT for 8 hours, (**d**) CuNWs kept at 150 °C for 8 hours, and (**e**) CuNWs kept at 300 °C for 8 hours.

The XRD data (Fig. 1) shows the evolution of Cu to Cu_2_O and CuO nanowires due to oxidation at different temperatures. Figure 1(a) shows a Cu nanowire pattern with crystalline peaks. The peaks positioned at 43.30, 50.48 and 74.13 perfectly match the 111, 200 and 220 planes. A Cu_2_O peak appears at the 110 plane, which may occur during the synthesis process in open air. Figure 1(b) shows the result of CuNWs where the temperature has been decreased to prevent oxidation. However, the formation of Cu_2_O still occurs due to the presence of water vapour, which causes Cu_2_O to form at the 110, 111, 200 and 220 planes^26^.

Figure 1(c) shows Cu_2_O peaks at the 110, 200 and 113 planes, and still Cu peaks at the 111, 200 and 220 planes for CuNWs kept at room temperature. Here CuO peaks begin to appear at 200 plane, which are absent for CuNWs kept at 0 °C. Figure 1(d) is dominated by the CuO and Cu_2_O peaks. The CuO phase appears at the 110, 200, 020 and 113 planes and Cu_2_O phase appears at the 110, 200, and 113 planes. Finally, Fig. 1(e) shows the detection of a large CuO peak at the 110, 200, 111, 112, 020 and 113 planes and Cu_2_O peaks are also detected at the 110, 200, 311 planes.

**Figure 2:**
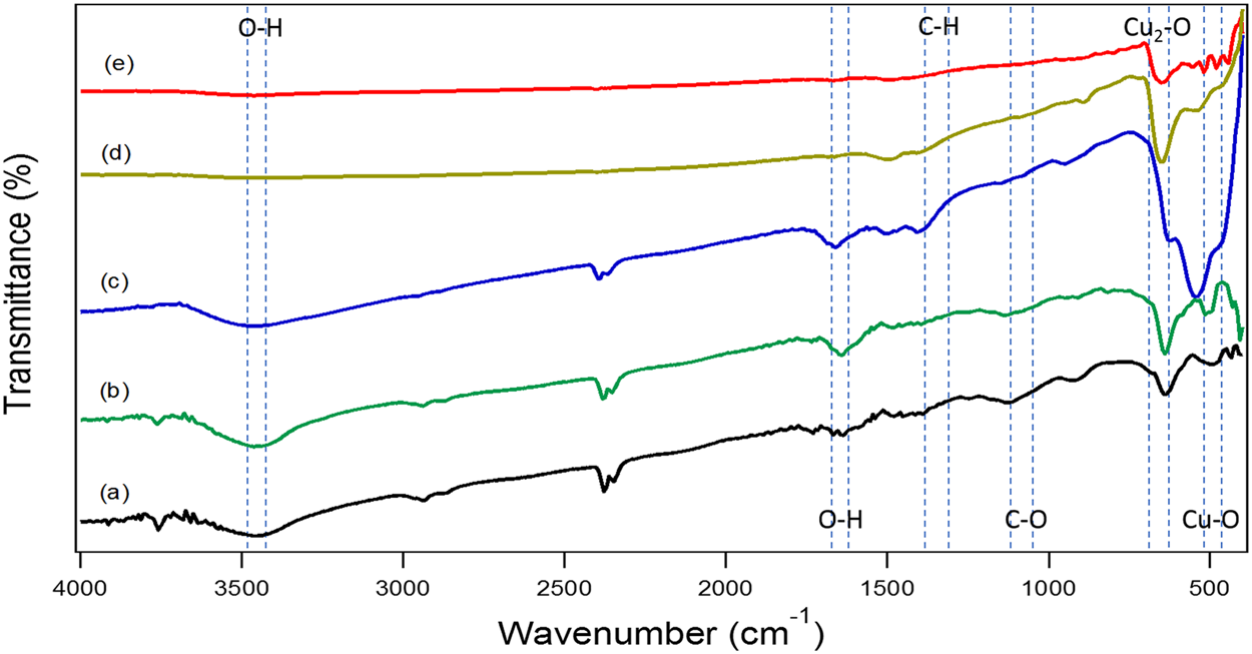
FTIR of Cu nanostructures. (**a**) CuNWs, (**b**) CuNWs kept at 0 °C for 8 hours, (**c**) CuNWs kept at RT for 8 hours, (**d**) CuNWs kept at 150 °C for 8 hours, and (**e**) CuNWs kept at 300 °C for 8 hours.

The FTIR spectrum (Fig. 2) was used to investigate the chemical structural of the CuNWs and the effects of oxidation. The high-frequency mode at 603.3 cm^−1^ may be attributed to the Cu-O stretching along the [101] direction, while the peak at 497 cm^−1^ can be assigned to the Cu-O stretching vibration along the [101] direction as observed in the reference^27^. Moreover, no other IR active mode was observed in the range of 605 to 660 cm^−1^, which totally rules out the existence of another phase, i.e. Cu_2_O^28^. The broad bands centred at 3426 and 1632 cm^−1^ are attributed to the O-H stretching and bending of the water^29^.

The weak band around 1384 cm^−1^ corresponds to the C-H vibration and absorption peak at 1116 cm^−1^ can be assigned to the C-O vibration coordinating to metal cations (Table 1)^29^.

**Table 1:** IR peaks and their assignments for CuNWs, Cu_2_O nanowires and CuO nanowires.

Assignment	Wavenumber cm^−1^				
	(a) CuNWs	(b) CuNWs (0 °C)	(c) CuNWs (25 °C)	(d) CuNWs (150 °C)	(e) CuONWs (300 °C)
Cu_2_-O	563.21	624.94	632.65	624.94	617.22
Cu-O stretching	493.77	486.06	478.35	509.21	493.54
O-H stretching	3356.13	3433.29	3417.72	3425.58	3425.58
O-H bending	1604.77	1627.92	1627.92	1634.64	1635.64
C-H	1342.45	1381.03	1357.34	1381.03	1404.18
C-O	1051.46	1126.43	1118.71	1064.71	1100.12

**Figure 3:**
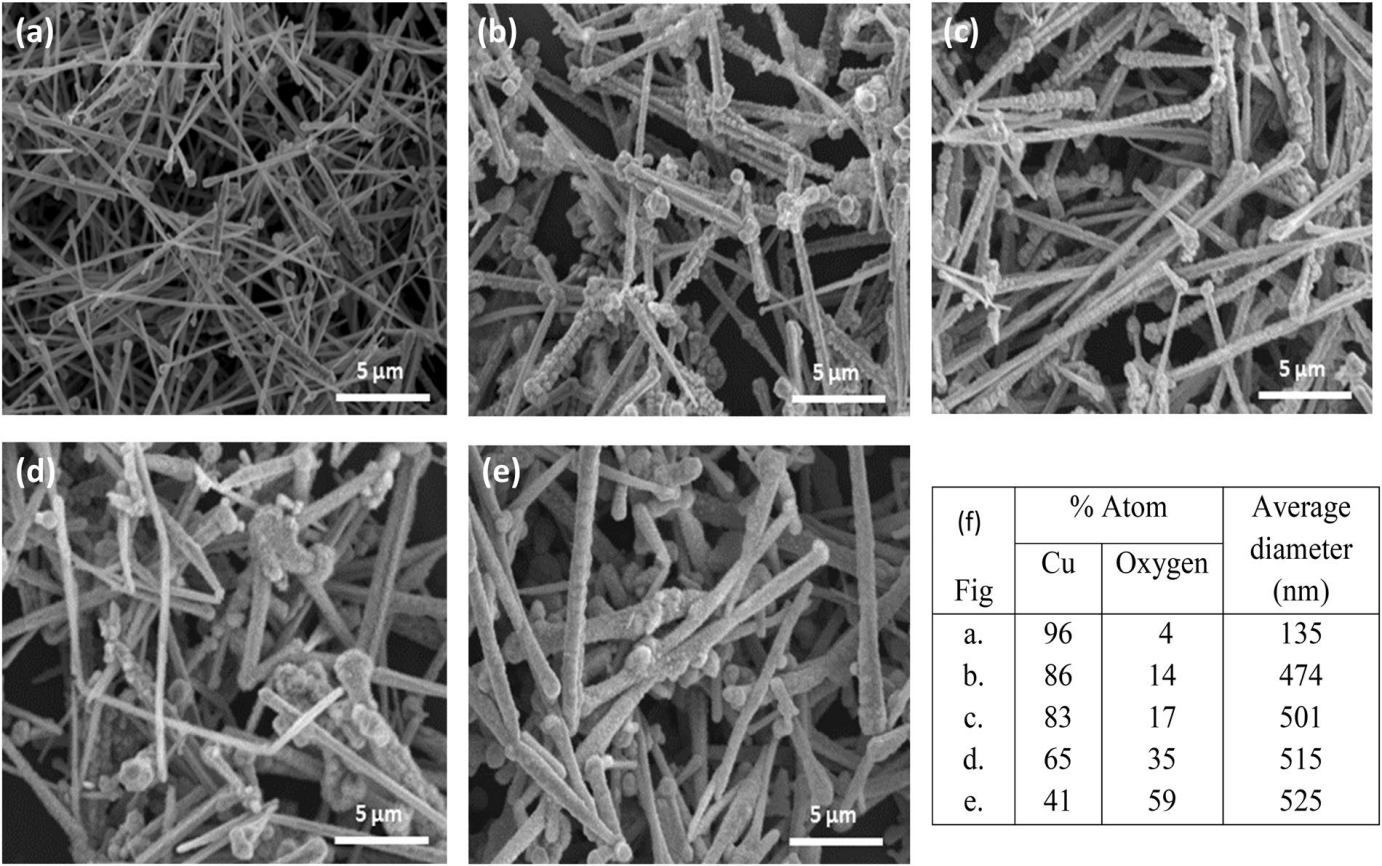
SEM images of the morphology of Cu nanostructures. (**a**) CuNWs, (**b**) CuNWs kept at 0 °C, (**c**) CuNWs kept at RT, (**d**) CuNWs kept at 150 °C, and (**e**) CuNWs kept at 300 °C. (**f**) The ratio of Cu and O in the atom after oxidation from EDX data and average diameter of the nanowires, calculated from the average diameter of 100 CuNWs.

Differences of the morphology of Cu nanostructures can be studied using SEM and TEM. Figure 3 shows SEM images for each temperature treatment. Figure 3(a) shows the morphology of CuNWs with an average diameter of 135 nm. In Fig. 3(b) the diameter of wires has increased to 474 nm due to oxidation and the absorption of water vapour creating Cu_2_O nanowires. This is because the oxygen concentration is less in the freezer than in ambient air. Water has a notable influence on the oxidation of Cu nanowires. This reaction can be described as follows in Eq. (1):

1 $$2{\rm{Cu}}+{{\rm{O}}}_{2}+{{\rm{H}}}_{2}\mathrm{O}\to {{\rm{Cu}}}_{2}\mathrm{O}+{{\rm{H}}}_{2}+{{\rm{O}}}_{2}{\rm{.}}$$

Figure 3(c) shows that although there was no significant difference to the morphology of CuNWs the average diameter of the Cu_2_O nanowires increased slightly to 501 nm for CuNWs at RT compared to 474 nm at 0 °C. This increase of diameter occurs due to oxidation which causes the nucleation of polyhedral crystals around the Cu nanowire. Although RT has a lower amount of water vapour than inside the freezer, it has a lot more free oxygen.

Figure 3(d,e) show the effects of annealing. Both images show a similar morphology, the wires become rough due to oxidation and the diameters increase further. The EDX data in Fig. 3(f), from the areas shown in the SEM images, shows the percentage of oxygen atoms at each temperature treatment. The data shows that the annealing treatment can increase the percentage of oxygen atoms in the nanowires. By increasing the temperature that CuNWs are treated at, from 0 °C to 300 °C, we can increase the amount of oxidation, i.e. the percentage of oxygen atoms in the structure, and simultaneously increase the average diameter of the nanowires.

**Figure 4:**
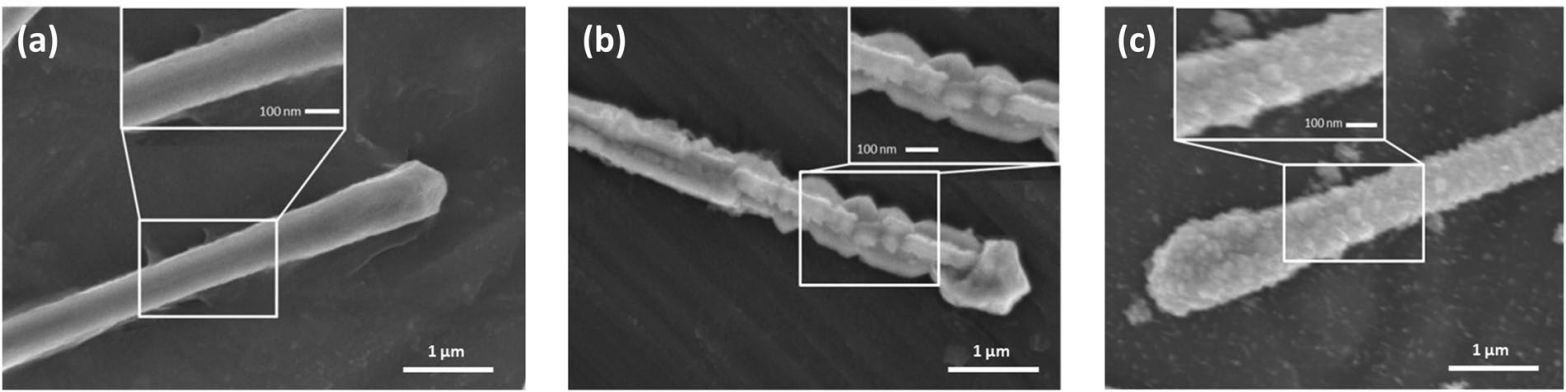
SEM images of the surface morphology of (**a**) a Cu nanowire, (**b**) Cu_2_O nanowire, and (**c**) CuO nanowire.

Annealing treatment also influences the morphology of CuNWs^17^. Figure 4 shows images of CuNWs before and after the annealing oxidation process. It is obvious to see that the morphology of the nanowires changes drastically.

**Figure 5:**
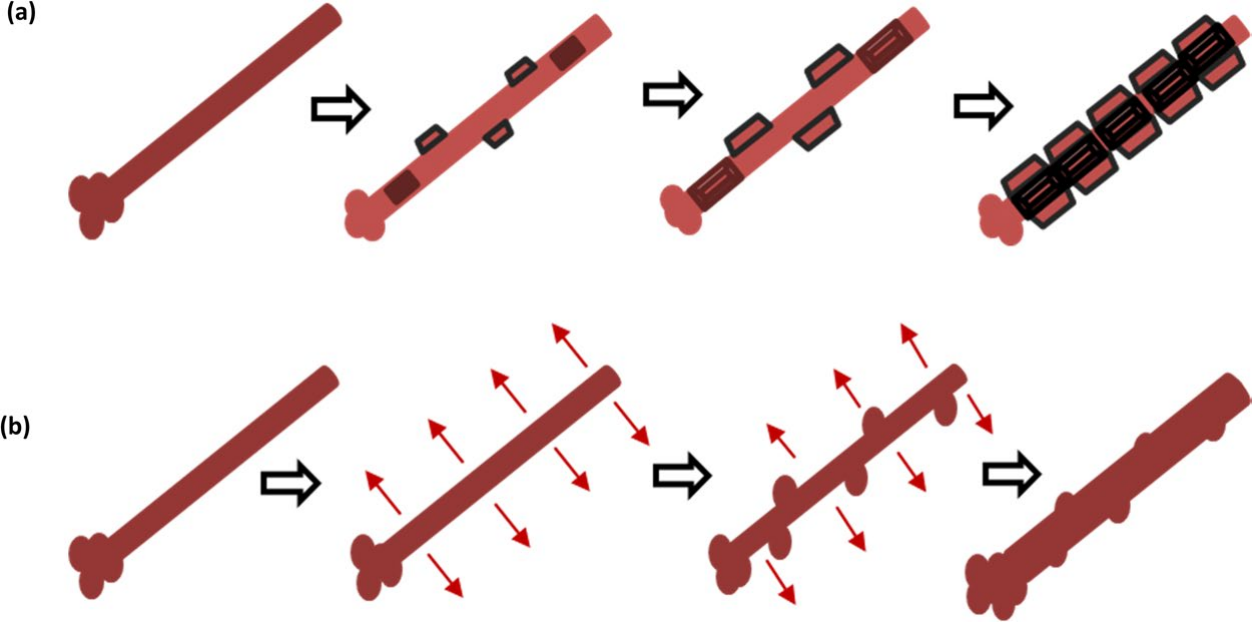
(**a**) Schematic of the effects of oxidation of a single Cu_2_O nanowire. (**b**) Schematic of the effects of oxidation of a single CuO nanowire.

Figure 4(a) shows that the surface of a pure, unoxidized CuNWs is uniform and smooth. In Fig. 4(b), the crystals nucleated around the nanowires and grew into polyhedral shapes forming crest effects along the length of the nanowire, and after annealing at a higher temperature (Fig. 4(c)) the surface of the CuNWs after oxidation becomes rough and is slightly damaged. This is due to the concentration of the oxygen atoms increasing during oxidation. The process can be described as shown in the schematic below (Fig. 5).

The oxidation process directly affects the surface structure and diameter of the nanowire. Figure 5(a) is a schematic of the oxidation process inducing nucleation of polyhedral crystals around the CuNWs. The metallic core grows and polyhedral crests form along the axis. Figure 5(b) shows a schematic of the oxidation process involving nucleation of a CuO nanowire. It is easy to see how the rough morphology along the axis develops. The nucleation causes an uneven increase in diameter, this affects the shape and morphology of CuNWs.

### Preventing the oxidation of Cu nanowires

**Figure 6:**
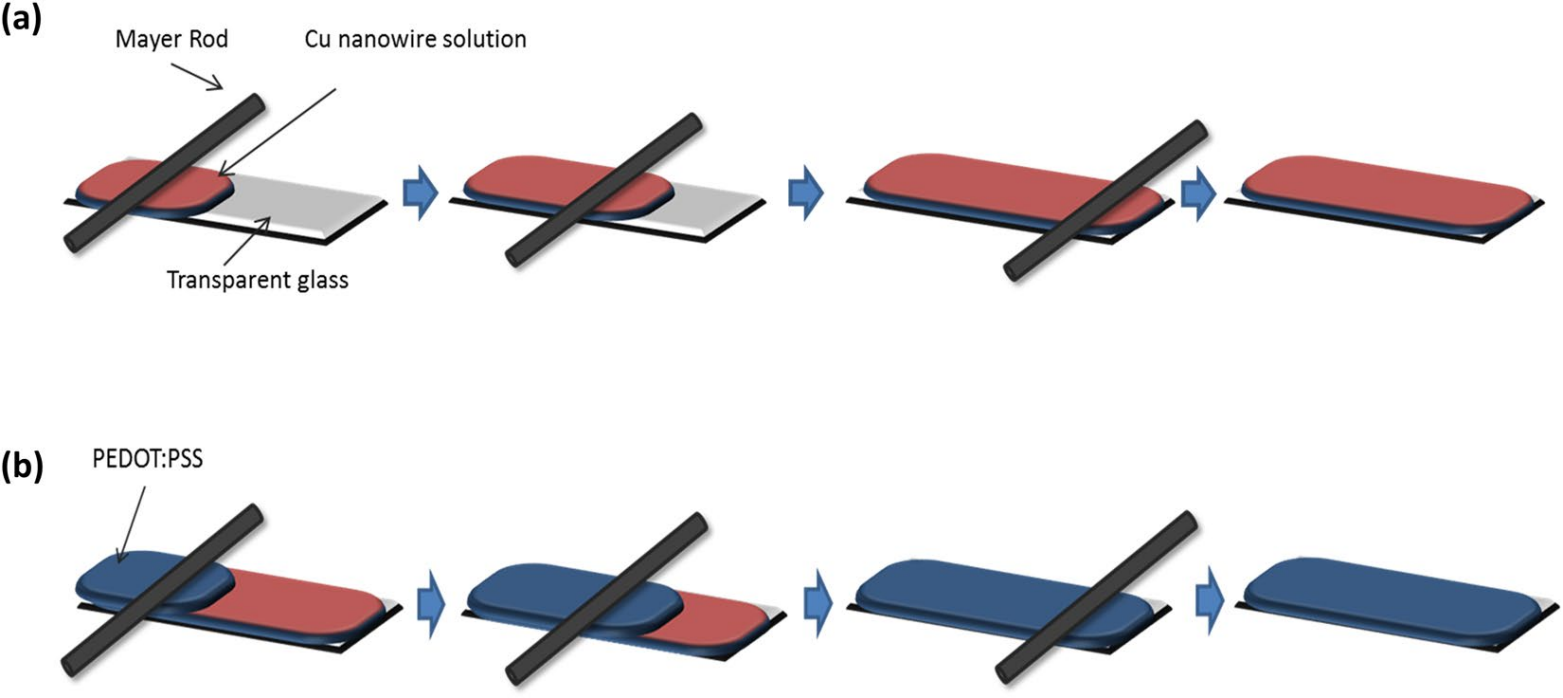
Schematic of the preparation of transparent conducting films using the Mayer rod method. (**a**) Transparent conducting film of CuNWs (**b**) Transparent conducting film of CuNWs with PEDOT:PSS coating.

Transparent conducting CuNWs films by were prepared using the Mayer rod method (Fig. 6). Using this method, we deposited a solution of CuNWs on the glass. Two types of transparent conducting films were prepared, one with only the CuNWs solution, and one with a capping layer of PEDOT:PSS over the nanowire solution. The films electrical properties were evaluated and compared using an IV-meter with a dual point probe.

**Figure 7:**
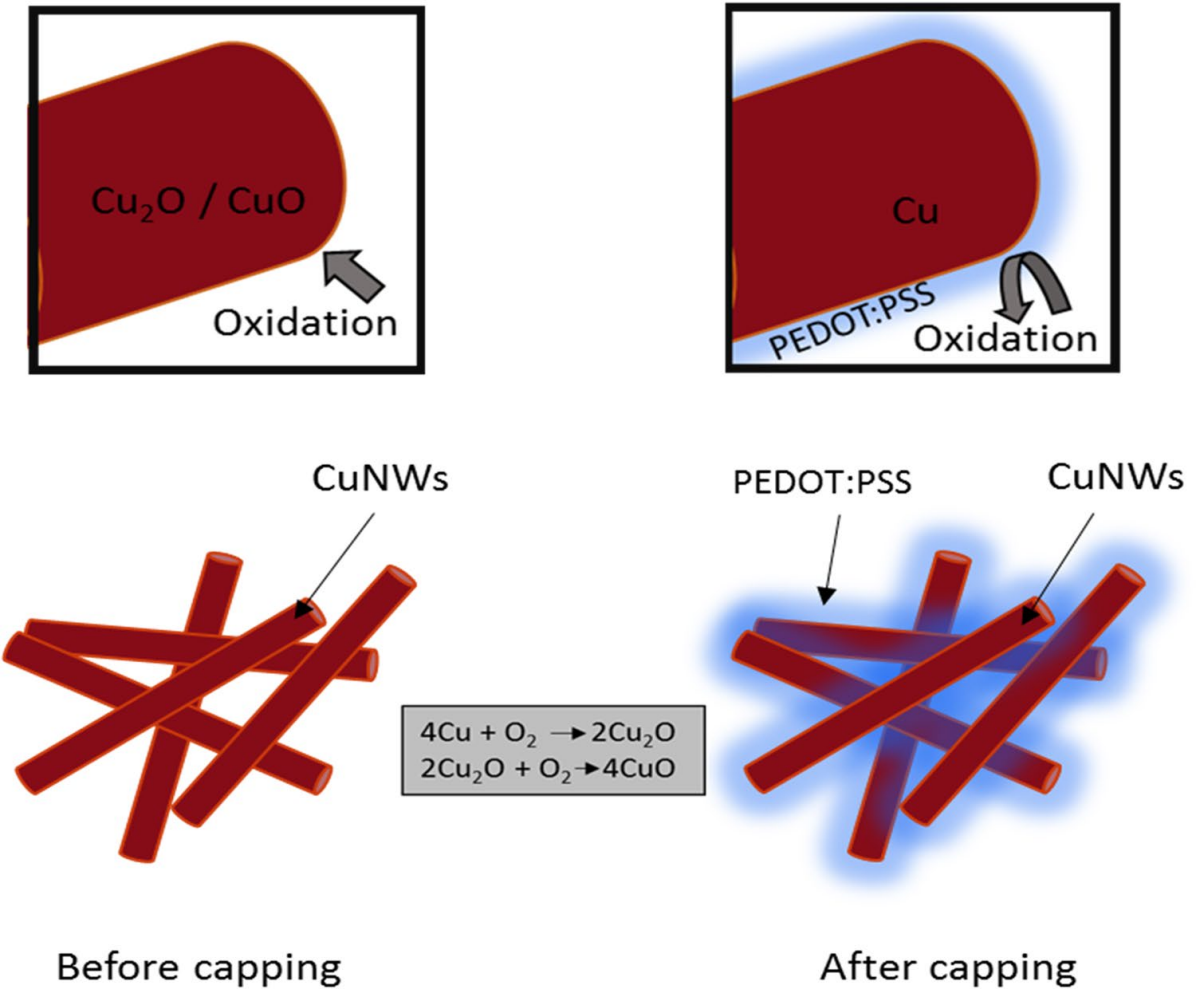
Schematic illustration of PEDOT: PSS capping layer protecting CuNWs from oxidation.

**Figure 8:**
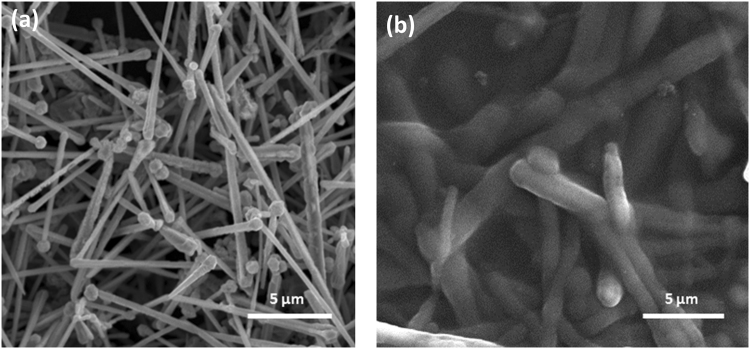
SEM images of (**a**) pure CuNWs and (**b**) the CuNWs coated with PEDOT:PSS.

Pure CuNWs are conductors before any kind of oxidation, but oxidation causes a splitting of the conduction and valence bands which creates an energy gap in the sample. Based on previous experiments and reports^7^, CuNWs without any form of capping will experience oxidation. When CuNWs are in contact with open air for some period of time, the band structure of the nanowire is disturbed by the oxygen. The presence of oxygen in Cu based structures create an energy band gap. When this happens, the characteristics of CuNWs change from that of a conductor to that of a semi-conductor, where the band gap increases as the amount of oxygen contamination increases. To protect the CuNWs from oxidation, we coated them with PEDOT:PSS, a material with conductive characteristics that can increase the conductivity of the CuNWs film, therefore negating the drawbacks of materials used previously i.e. PVP and EDA. Figure 7 shows a schematic illustration of the capping process and Fig. 8 shows SEM images of the morphology of CuNWs with and without the PEDOT:PSS coating.

In Fig. 8(a), the pure nanowires average diameter without PEDOT:PSS is 120 nm with lengths of several µm, while after the coating of PEDOT:PSS (Fig. 8(b)), the diameter increases with no change in length, as one would expect. The role of PEDOT:PSS on the CuNWs film also results in a better connection between the nanowires themselves and a strong bond between the nanowires and the substrate. This increased quality of connection serves to help the efficiency of electron transfer on the CuNWs film.

**Figure 9:**
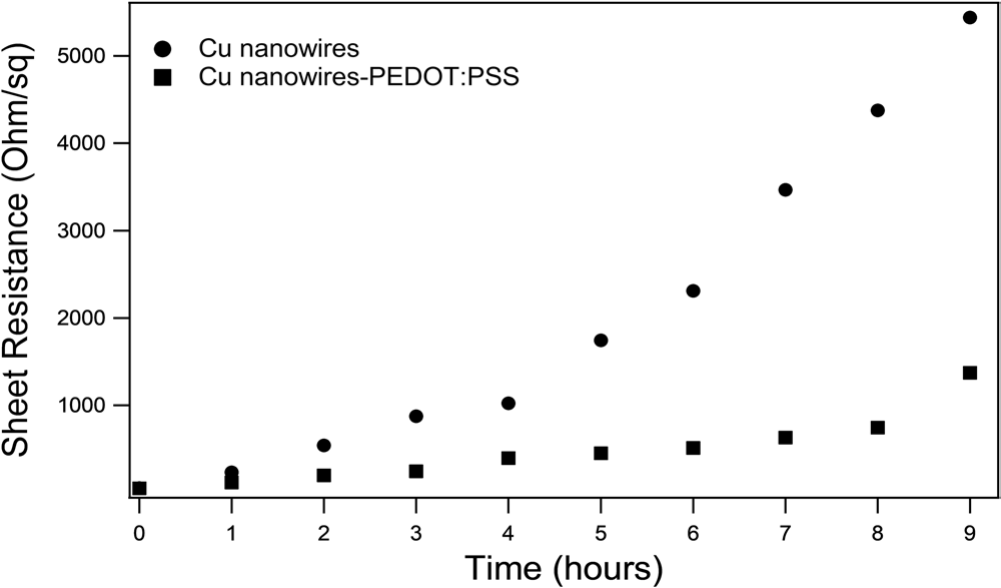
Sheet resistance over time for CuNWs and CuNWs coated with PEDOT:PSS.

The pure CuNWs were sensitive to oxygen and environmental contamination. We applied the PEDOT:PSS coated CuNWs to a transparent conducting electrode and measured the sheet resistance each hour over a period of 9 hours. Figure 9 shows that after 2 hours, oxidation has already taken place and sheet resistance has already started to increase for CuNWs. Whereas over the same time period the CuNWs coated with PEDOT:PSS show a steady and almost constant resistance, and continue to resist oxidation and increased resistance for around 6 hours. These results clearly indicate that the big challenge encountered by CuNWs electrodes is their instability due to oxidation. This problem could be resolved using a PEDOT:PSS coating on the CuNWs films. The flexibility of metal nanowire-based electrodes is an outstanding benefit compared to ITO and this feature is maintained by CuNWs:PEDOT:PSS composite films.

## Discussion

The effects and prevention of oxidation of CuNWs was successfully investigated. The amount of oxidation varied based on temperature. After analysing CuNWs kept at four different temperatures the results are as follows. Keeping the samples at 0 °C produces Cu_2_O nanowires with 14% of oxygen atoms. CuNWs kept at RT produces Cu_2_O nanowires with 17% oxygen atom contents. Annealing the nanowires in an oven at 150 °C and 300 °C produces CuO nanowires with 35% and 59% of oxygen atoms respectively. We successfully used PEDOT:PSS as a coating to protect CuNWs from oxidation. CuNWs:PEDOT:PSS has a stable and consistent conductivity for up to eight hours, a four-fold increase on pure Cu nanowires. The strategy developed in this study is beneficial to future work for applications of CuNWs in the field of transparent conducting electrodes, sensors and modern electronic devices.

## Methods

### Materials

We used (Cu(NO_3_)_2_.3H_2_O) (99%, Merck) as the main material, NaOH (99%, Merck) as the pH adjusting element, ethylenediamine (EDA, Merck) as the capping agent, hydrazine (N_2_H_4_, Merck) as a reductant and Poly(3,4-ethylenedioxythiophene) polystyrene sulfonate (PEDOT:PSS, PH 1000, heraeous) as a protective oxidation.

### Synthesis of Cu nanowires

The CuNWs were synthesized using the aqueous solution method to produce CuNWs at a large scale^4^. Cu (II) nitrate trihydrate was used as the raw material, EDA as the capping agent and hydrazine as the reductant. Cu (NO_3_)_2_.3H_2_O (20 mL, 0.025 M) was mixed with NaOH (100 mL, 15 M) in the 150 mL reaction flask. The colour of the solution was deep blue which indicates the formation of (Cu(OH)_4_)^−2^. After 2 minutes, 0.5 mL of EDA and 0.25 mL of hydrazine (35. wt. %) was added. After the addition of hydrazine, the colour turned clear blue. 15 minutes after the hydrazine was added, CuNWs started to form. Cu nanoparticles formed first, then over time, CuNWs formed. 60 minutes later, CuNWs floated to the top of the solution. During the whole process, the mixtures were stirred at 60 rpm.

### Oxidation of Cu nanowires

**Figure 10:**
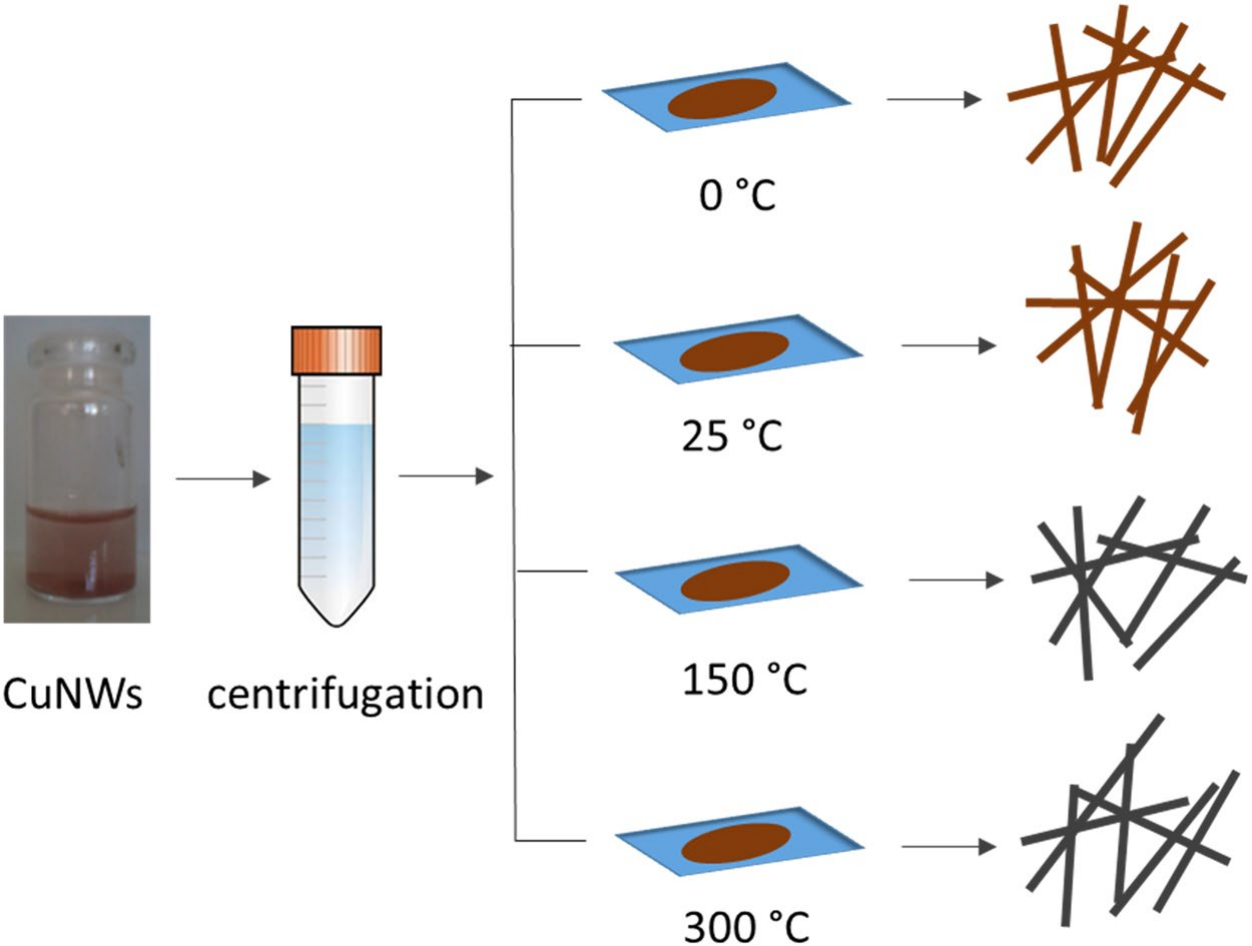
Schematic the oxidation of CuNWs with the 4 temperature treatments.

After the synthesis of CuNWs, the product was washed with ethanol and then centrifuged 5 times at 10,000 rpm, for 10 minutes. The samples were then transferred to 4 different temperature treatments for 8 hours: (1) in a freezer (0 °C), (2) at room temperature (RT) (25 °C), (3) in an oven (150 °C), and (4) in an oven at 300 °C (Fig. 10). The individual solid precipitates were collected for the analysis.

The aim of the four different heating treatments was to investigate the effect of temperature on the oxidation of Cu nanowires. The two-step oxidation process can be described as follows in Eqs (2) and (3):

2 $$4{\rm{Cu}}+{{\rm{O}}}_{2}\to 2{{\rm{Cu}}}_{2}{\rm{O}}{\rm{.}}$$

3 $$2{{\rm{Cu}}}_{2}{\rm{O}}+{{\rm{O}}}_{2}\to 4{\rm{CuO}}.$$

The effects of oxidation were analysed using the X-ray diffraction (XRD, Shimadzu-6000) patterns of the samples Cu_2_O and CuO peaks. Fourier Transform Infrared Spectroscopy (FTIR, Shimadzu FTIR-8010 PC) was used with wave number of 400–4000 cm^−1^ to investigate the Cu-O stretching and bending. Furthermore, Scanning Electron Microscopy-Energy Dispersive X-ray Spectroscopy (SEM-EDX, JEOL JSM-6510) was used to study the effects of temperature on the morphology and element distribution of the CuNWs.

### Preventing the oxidation of Cu nanowires

The oxidation of CuNWs can be prevented by capping^25^. Capping functions to block oxygen contamination from the environment by encapsulating the nanowires with a material. As previously discussed, up to now, the capping materials have resulted in a loss of conductivity and therefore usefulness of CuNWs. To combat this, we used a coating of PEDOT:PSS on the CuNWs with a ratio of PEDOT:PSS to Cu nanowire of 50:50 in a volume of 1 mL. PEDOT:PSS has advantages over PVA and EDA coatings as it is a conductive polymer, allowing for the protection against oxidation without the reduction of conductivity, making it a great choice for transparent conducting electrodes.

### Preparation of the Cu nanowire film and analysis of electrical properties

Films of Cu nanowires were prepared in 2 mL of ethanol, then deposited using the Meyer rod coating method. Meyer rod coating is a more easily scalable method to produce transparent films of metal nanowires^30^ compared to methods such as spray coating, as it enables faster deposition of nanowires to the film and allows the distribution of CuNWs to be controlled. To investigate oxidation of CuNWs and its prevention, we analysed the electrical properties of films with and without the PEDOT:PSS coating, using an I-V Meter (Keithley 2041) with a dual-point probe to compare the sheet resistance between CuNWs every hour for 9 hours.
